# Generating EQ-5D-5L health utility scores from BASDAI and BASFI: a mapping study in patients with axial spondyloarthritis using longitudinal UK registry data

**DOI:** 10.1007/s10198-022-01429-x

**Published:** 2022-02-03

**Authors:** Aileen R. Neilson, Gareth T. Jones, Gary J. Macfarlane, Ejaz MI Pathan, Paul McNamee

**Affiliations:** 1 Edinburgh Clinical Trials Unit (ECTU), Usher Institute, University of Edinburgh, Edinburgh, UK; 2 Epidemiology Group, Institute of Applied Health Sciences, School of Medicine, Medical Sciences and Nutrition, University of Aberdeen, Aberdeen, UK; 3 Rheumatology Department, Freeman Hospital, Newcastle upon Tyne Hospitals NHS Foundation Trust, Newcastle upon Tyne, UK; 4 Health Economics Research Unit, Institute of Applied Health Sciences, School of Medicine, Medical Sciences and Nutrition, University of Aberdeen, Aberdeen, UK

**Keywords:** Mixture models, Utility mapping, Response mapping, EQ-5D-5L, BASDAI/BASFI, Axial spondyloarthritis

## Abstract

**Background:**

Preference-based health-state utility values (HSUVs), such as the EuroQol five-dimensional questionnaire (EQ-5D-5L), are needed to calculate quality-adjusted life-years (QALYs) for cost-effectiveness analyses. However, these are rarely used in clinical trials of interventions in axial spondyloarthritis (axSpA). In these cases, mapping can be used to predict HSUVs.

**Objective:**

To develop mapping algorithms to estimate EQ-5D-5L HSUVs from the Bath Ankylosing Disease Activity Index (BASDAI) and the Bath Ankylosing Spondylitis Functional Index (BASFI).

**Methods:**

Data from the British Society for Rheumatology Biologics Register in Ankylosing Spondylitis (BSRBR-AS) provided 5122 observations with complete BASDAI, BASFI, and EQ-5D-5L responses covering the full range of disease severity. We compared direct mapping using adjusted limited dependent variable mixture models (ALDVMMs) and optional inclusion of the gap between full health and the next feasible value with indirect response mapping using ordered probit (OPROBIT) and generalised ordered probit (GOPROBIT) models. Explanatory variables included BASDAI, BASFI, and age. Metrics to assess model goodness-of-fit and performance/accuracy included Akaike and Bayesian information criteria (AIC/BIC), mean absolute error (MAE) and root mean square error (RMSE), plotting predictive vs. observed estimates across the range of BASDAI/BASFI and comparing simulated data with the original data set for the preferred/best model.

**Results:**

Overall, the ALDVMM models that did not formally include the gap between full health and the next feasible value outperformed those that did. The four-component mixture models (with squared terms included) performed better than the three-component models. Response mapping using GOPROBIT (no squared terms included) or OPROBIT (with squared terms included) offered the next best performing models after the three-component ALDVMM (with squared terms). Simulated data of the preferred model (ALDVMM with four-components) did not significantly underestimate uncertainty across most of the range of EQ-5D-5L values, however the proportion of data at full health was underrepresented, likely due in part to model fitting on a small number of observations at this point in the actual data (4%).

**Conclusions:**

The mapping algorithms developed in this study enabled the generation of EQ-5D-5L utilities from BASDAI/BASFI. The indirect mapping equations reported for the EQ-5D-5L facilitate the calculation of the EQ-5D-5L utility scores using other UK and country-specific value sets.

**Supplementary Information:**

The online version contains supplementary material available at 10.1007/s10198-022-01429-x.

## Introduction

The EuroQol five-dimensional questionnaire (EQ-5D) is one of the most frequently used measures for the collection of health-related quality of life (HRQoL) data, required for use in cost-effectiveness analysis when quality-adjusted life-years (QALYs) are calculated. In the UK this is reflected in current National Institute of Health and Care Excellence (NICE) Methods Guidance [1] relating to economic evaluation. However, the guidance also recognises that outcomes included in clinical studies often do not include preference-based measures (PBMs) such as the EQ-5D to generate health-state utility values (HSUVs). In such cases, when PBM data are unavailable, “mapping” from other relevant clinical outcome and disease measures that have been collected in clinical trials to estimate a PBM has been advocated [e.g., 2, 3]. In a review of economic models included in 71 NICE submissions (53 manufacturer submissions, 18 assessment group reports) the EQ-5D was used in 49% of submissions and mapping to a generic HRQoL measure was performed in 27% of submissions [4].

Axial spondyloarthritis (axSpA) is a common rheumatic chronic progressive inflammatory disease, leading to joint damage/pain, stiffness, impaired physical function, fatigue and reductions in quality of life [5]. AxSpA primarily affects the spine and sacroiliac joints but it also associated with peripheral arthritis plus various extra-articular features such as enthesitis, uveitis, inflammatory bowel disease and psoriasis. AxSpA typically has its onset early in adulthood [6] and is more common in men than in women [7]. AxSpA patients incur significant direct NHS costs, which are mainly due to costs associated with prescriptions, and outpatient and day unit use [8]. There are also indirect costs to society as axSpA affects young patients who have to take time off work and in the worst cases may permanently cease paid employment [9–11]. Until recently, treatment has largely been limited to NSAIDS and physiotherapy, while DMARDs, though effective in other inflammatory conditions, have shown little efficacy for axSpA. The introduction of anti-TNF biologic therapies licensed for use in the management of axSpA (e.g., etanercept, adalimumab) has been associated with significantly improved outcomes, including improvements in pain, stiffness, fatigue, and work outcomes such as improved productivity [12–16]. However, many of the pivotal trials for new biologics therapies did not include a generic preference-based HRQoL instrument such as the EQ-5D nor a preference-based condition-specific HRQoL measure. These preference-based measures allow direct comparison of outcomes associated with alterative interventions both within similar and across different therapeutic areas. Within the field of axSpA, analysts have conducted mapping studies to estimate the relationship between EQ-5D and clinical outcome measures, most predominantly the Bath Ankylosing Disease Activity Index (BASDAI) [17] and the Bath Ankylosing Spondylitis Functional Index (BASFI) [18]. In the UK for example, all appraisals of biologic therapies undertaken by NICE and their broader guidelines on the management of axSpA relied on a mapping approach [19]. Until recently, such mapping studies were based on linear regression models [19–24]. However, recent evidence suggests that such an approach may lead to biased estimates, with such models tending to show poor statistical properties in terms of goodness-of-fit, with a major limitation being lack of ability to predict values at the extreme ends of the distribution [e.g., 25, 26]. In particular the statistical model underestimates utility values for those patients with little or no functional disability (and/or low disease activity) but overestimates the utility score for those with poor function (and/or high disease activity). Ultimately, this may lead to biased estimates of the cost-effectiveness of interventions. This poor statistical fit typically arises due to the special characteristics of health utility instruments. Specifically, utility instruments have upper and lower bounds, tend to have a mass at one or more at the upper bounds, and can be further characterised by multimodality and skewness. Of course, we are not implying that researchers should not ever consider using these ‘basic’ models – only to flag an awareness that linear models have been shown to perform less well relative to other mapping approaches in the published literature. Many published mapping studies do in fact include the results from linear regression models, to allow comparison with other mapping approaches and so we also have done this in the current mapping study.

Recently Wailoo et al. [27] implemented an alternative mapping approach that linked BASDAI and BASFI to the 3-level version of the EQ-5D (EQ-5D-3L). This used a mixture modelling approach, which involves mixtures of normal distributions being used because of their flexibility and ability to capture multimodality [28, 29]. This approach does not permit generation of predicted values outside the feasible range by its design. This demonstrated improved statistical performance over the linear regression model in terms of Akaike and Bayesian information criteria, root mean square error, and mean absolute error, fitted the data well at high EQ-5D levels and did not predict unfeasible EQ-5D values. This approach has also been applied in a number of different diseases and conditions [30] including rheumatoid arthritis (RA) [31, 32] and asthma [25], breast cancer populations [30], and traumatic brain injury [33].

In axSpA, few studies of directly elicited EQ-5D data have been published, and as a consequence, economic models included in Health Technology Assessments (HTAs) of axSpA treatments have been based on EQ-5D utilities estimated from mapping exercises [19] using the EQ-5D-3L. For instance, three studies identified in the HERC Database of Mapping Studies [34] are all based on the EQ-5D-3L [21, 27, 35]. Two studies used simple linear regression statistical model mapping methods [21, 35], a feature common to all previous NICE assessments of biologic therapies in axSpA [19]. A third study found that other direct mapping methods using bespoke adjusted limited dependent variable mixture models (ALDVMMs) and indirect response mapping offered a better fit to the observed data than simple linear regression [27]. To our knowledge, there are currently no published mapping algorithms that estimate EQ-5D-5L utilities from BASDAI or BASFI scores. This paper provides the first mapping algorithms from BASDAI/BASFI to the EQ-5D-5L instrument for axSpA. Our study is also the first mapping study that uses a UK data set based on data from the British Society for Rheumatology Biologics Register in AS (BSRBR-AS) which includes observations that come from England, Scotland and Wales (with the largest contribution from England, *n* = 4595, and *n* = 382, *n* = 145, respectively). We applied the current England published tariff value set which has been used in a number of recent studies in the mapping literature [e.g., 25, 36–38], although we acknowledge that NICE currently recommend mapping to the 3L using the van Hout crosswalk to convert the EQ-5D-5L responses to EQ-5D-3L scores.

## Methods

### Study dataset

Patient-level data were collected over a 5-year period from December 2012 to June 2017 as part of a UK-wide prospective observational cohort study – the British Society for Rheumatology Biologics Register in AS (BSRBR-AS). For a full description of the study, see Macfarlane et al. [39]. The study included patients with axSpA who were naïve to biologic therapy at the time of recruitment. Those about to commence anti-TNF biologic therapy entered a “biologic” sub-cohort with other patients assigned to a “non-biologic” sub-cohort. The primary objective of the study was to determine the long-term safety of biologic treatment. Secondary objectives were to assess differences in malignancy, serious comorbidity, all-cause mortality, and impact on specific clinical domains (physical and mental health and QoL) including work outcomes between biologic and non-biologic cohorts. Patients were followed-up for up to 5 years. Data were obtained at baseline and at standard clinical follow-up visits – at 3, 6, and 12 months and then annually for the biologic cohort, and annually for the non-biologic cohort. Each follow-up involved the collection of clinical and self-report data (BASDAI [17], BASFI [18], and the EQ-5D-5L [40, 41]. Patients were potentially eligible for the BSRBR-AS study if they were aged ≥ 16 years and met the Assessment of SpondyloArthritis Society (ASAS) criteria for radiographic and non-radiographic axSpA.

### Outcome measures

#### EQ-5D-5L

The EQ-5D-5L is a generic HRQoL instrument comprising five dimensions (mobility, self-care, usual activities, pain/discomfort, and anxiety/depression) [41]. There are five-levels of the perceived problems per dimension (no problems, slight problems, moderate problems, severe problems, and extreme problems). Responses to the questionnaire were scored using the English EQ-5D-5L value set [42] which ranges from an ‘index score’ of –0.285 (state 55555) to 1 (state 11111). A value of 1 represents full/perfect health, a value of 0 is considered equivalent to being dead, and values can also be negative, representing a state worse than death. There is also a gap between full health and the next feasible health state value of 0.950, which is referred to as the truncation point [25, 30]. This is the highest possible value generated by the EQ-5D-5L instrument that is not represented by full health. The lower limit for the EQ-5D-5L is − 0.285.

#### BASDAI

The BASDAI is a self-reported instrument consisting of six questions focusing on five major symptoms of the disease over the past week (fatigue, spinal and hip pain, swelling and pain in peripheral joints, enthesitis, duration and severity of morning stiffness) [17]. The BASDAI consists of a 1 – 10 scale (1 = no problem and 10 = the worst problem). To give each symptom equal weighting, the mean (average) of the two scores relating to morning stiffness is taken. The resulting 0 to 50 score is divided by 5 to give a final 0 – 10 BASDAI score.

#### BASFI

The BASFI is a self-assessed instrument represented as a mean of 10 questions from a visual analogue scale (ranging from 0 being easy and 10 being impossible), 8 of which relate to the person’s degree of functional limitation and 2 of which relate to a subject’s ability to cope with everyday life/tasks [18]. Each question is answered on a 10 cm horizontal visual analogue scale, the mean of which gives the BASFI score (0–10). An increase in BASFI score indicates a worsening condition.

### Statistical analysis

#### Conceptual overlap

The estimation of a mapping algorithm relies on conceptual overlap between the source measure/s (BASDAI/BASFI) and the target measure (EQ-5D-5L). No overlap in content implies that mapping is unlikely to capture the relationship between the measures to estimate health utilities [43]. Spearman rank correlation coefficients were used to test the correlations between the BASDAI/BASFI total scores and EQ-5D-5L index scores or item responses/domains, to investigate the degree of conceptual overlap.

### Model development

Our study followed published recommendations of mapping best practice [3, 44–46]. Generally, there are two broad approaches to mapping: direct mapping, which models the EQ-5D index values using regression models, and indirect mapping, also referred to as response mapping, which models responses to each item of EQ-5D and then calculates the predicted utilities as a separate second step. We tested both approaches. We also included linear regression for comparative purposes with these two methods.

In the direct mapping approach, adjusted limited dependent variable mixed models (i.e., direct mapping models) were implemented using the publicly available Stata command “aldvmm” [47]. This method for estimating EQ-5D has been previously implemented by Hernández et al. [e.g., 28, 29], who combined bespoke distributions in a mixture model. These include a discrete element using multinomial logit models for the probability of component (or latent class) membership, including a component representing full health. Using multiple components in a mixture model allows us to compare the multimodal properties of the distribution. The EQ-5D-5L does not show a (single) normal distribution and so it is important to be able to accurately estimate this unusual distribution. The ALDVMM method limits the underlying distribution which is limited at both ends, with a gap and allows a mass of observations at one end like a Tobit model. The mixture of several of these allows non-normal distributions to be approximated.

By limiting the dependent variable, the ALDVMM prevents prediction below -0.285 or above 1. It also takes into account the next feasible health state (i.e., to mirror feasible values in the tariff). The aldvmm Stata function has extra options including permitting the user to specify the next feasible value after full health for the target utility instrument (i.e. the “truncation point,” thus creating the typical gap seen in PBMs). There is also the possibility to specify no truncation and therefore allow each component of the mixture model to be fully continuous up to the highest feasible value of 1 for full health [25, 30]. This modelling approach has also been successfully applied in previous mapping studies (to the EQ-5D-3L) in a range of settings, including axSpA, and RA populations in the UK setting [27, 30, 31].

We tested models with different numbers of components. In particular, we considered models with three and four separate components, informed on the basis of existing mapping literature [27–30]. We further compared different numbers of components in the mixture models with and without a specified gap between full health and the next feasible value.

In the response mapping approach, ordered probit (OPROBIT) and generalised ordered probit (GOPROBIT) models were estimated for each dimension/domain of the EQ-5D-5L to predict the probabilities of a given response level. We then calculated the expected EQ-5D-5L scores on the basis of the probabilities of each of the possible 3125 health states as a second step. This modelling approach has been successfully applied to the EQ-5D-3L previously in AS [27] and RA populations in the UK setting [29, 31].

All analyses were undertaken with STATA v15. Models were estimated using maximum likelihood. Due to individuals contributing multiple observations, robust standard errors (and their reported *p* values) were estimated to take account of the repeated observations, similar to previous approaches [27]. In terms of model specification, we considered BASDAI, BASFI, and age, as potential independent variables (and squared terms) in all models, as these are the most common covariates which have been previously included in published mapping models using the EQ-5D-3L [19, 27].

### Assessing model performance

Good mapping practice suggests that mapping algorithms should, where possible, be estimated in an ‘estimation sample’ and then predictions should be made in an external ‘validation sample’ [44]. Predicted scores are then compared with actual observed scores to assess model performance. In our study however, no such suitable external data set was available and therefore we used the full data (5122) to estimate and validate model predictions. This approach is consistent with the approach used in the previous mapping study using the EQ-5D-3L in AS [27].

To compare results across models/ assess predictive ability we considered different measures of goodness-of-fit statistics in line with good mapping best practice [3, 44–46]. These comprised Akaike’s and Bayesian Information Criteria (AIC/BIC), overall mean estimates, mean error (ME), mean absolute error (MAE), and root mean squared error (RMSE), as well as considered visual representations of model fit. We plotted the means of the predicted values of EQ-5D-5L with the mean observed values, across the range of disease severity. Also, for those settings in which analysts wish to use the models to simulate individual level EQ-5D data, rather than generate expected (e.g., mean cohort) EQ-5D values, we then additionally compared the simulated EQ-5D-5L data for the preferred model with the original actual/observed data. This additional step allows assessment of how well the chosen model will estimate any uncertainty in the model. This further level of model validation is important, as often cost-effectiveness analyses using a long-time horizon are performed by simulating many hypothetical, individual patients. In this situation, the analyst requires statistical models to estimate EQ-5D scores for these individuals. The simulated data are produced by incorporating not only the explanatory variables for each patient but also the random error term(s) for the statistical model. To illustrate the predictive accuracy in terms of uncertainty of the preferred model, we simulated 1000 data points from the preferred model for each of the observations in the BSRBR-AS dataset and then plotted the cumulative distribution function (CDF).

Models were ranked according to their ME, MAE, and RMSE, such that each model had a set of three rankings. The three rankings were summed to calculate an overall ranking. The lower the overall ranking, the better the performance of the model, i.e., thus, in principle, the best performing one model would be the lowest value in the overall ranking results.

To enable future use in economic evaluations, an Excel tool to implement selected algorithms was produced. Also, variance–covariance matrices were included with the spreadsheet tool for the purpose of probabilistic sensitivity analysis [3, 44–46].

## Results

### Descriptive statistics

**Fig. 1:**
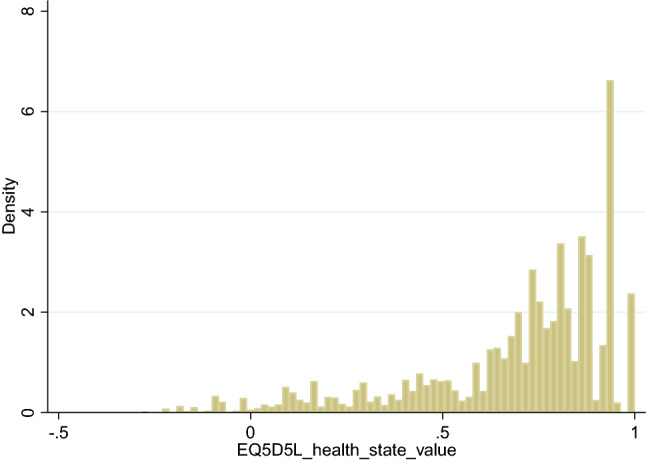
Distribution of EQ-5D-5L values (Total sample, *N* = 5122)

The BSRBR-AS mapping dataset of patients had complete BASDAI and BASFI and complete EQ-5D-5L data. There were no patients excluded from the current mapping study. This included a total of 5122 observations with complete information on BASDAI, BASFI and EQ-5D-5L from 1965 patients. Patients’ responses spanned the full BASDAI and BASFI values. For the EQ-5D-5L 4.4% (223/5122) of observations were at full health (i.e., values = 1.0) and 2.8% (144/5122) of observations were negative. Figure 1 shows a histogram of the distribution of EQ-5D-5L index values/scores, exhibiting several of the usual characteristics associated with this measure. First there is a mass of observations at 1. Second, there is a gap (where no EQ-5D values are possible) between these observations and those for any level of impairment, e.g., the gap in values between 1 and 0.950 (next best health state value). The data show a large number of observations clustered around the value of 0.95, specifically: 0.2% of observations had values of 0.95 (*n* = 11); 0.3% of 0.942 (*n* = 17); 11.8% of 0.937 (*n* = 606), and 2.4% (*n* = 124) were in the range 0.90 < 0.924. In addition, there is a multi-modal distribution (at least 2 peaks).

Patient characteristics and summary statistics are reported in Table 1. Overall, the mean (SD) age was 51.0 (14.5) years old and the majority of respondents were male (70.0%). The mean (SD) BASDAI and BASFI scores were 4.3 (2.5) and 4.4 (2.9), respectively. The mean EQ-5D value was 0.69 (0.26).

**Table 1:** Sample characteristics

Variable	*n* = 5122	Mean (± SD)/Range (min, max)
Age (years)	5122	51.0 ± 14.5 17.4, 100.0
BASDAI (0–10)	5122	4.3 ± 2.5 0, 10
BASFI (0–10)	5122	4.4 ± 2.9 0, 10
EQ-5D-5L index value	5122	0.693 ± 0.26 − 0.285, 1
		%
Gender: male (%)	3588	70.05
EQ-5D-5L profile		
Mobility		
No problems	1951	38.1
Slight problems	1425	27.8
Moderate problems	1175	22.9
Severe problems	544	10.6
Unable	27	0.5
Self-care		
No problems	3147	61.4
Slight problems	1046	20.4
Moderate problems	707	13.8
Severe problems	200	3.9
Unable	22	0.4
Usual activities		
No problems	1646	32.1
Slight problems	1698	33.2
Moderate problems	1168	22.8
Severe problems	514	10.0
Unable	96	1.9
Pain and discomfort		
No	351	6.85
Slight	2038	39.60
Moderate	1706	33.31
Severe	818	15.97
Extreme	209	4.08
Anxiety and depression		
Not	2305	45.00
Slightly	1586	30.96
Moderately	924	18.04
Severely	232	4.53
Extremely	75	1.46

The proportion of observations at: full health = 4.4% (*n* = 223), 0.95 = 0.2% (*n* = 11), 0.942 = 0.3% (*n* = 17), 0.937 = 11.8% (*n* = 606), 0.924 = 0.03% (*n* = 2), 0.922 = 0.8% (*n* = 43), 0.916 = 1.6% (*n* = 81), negative = 2.8% (*n* = 144)

*BASDAI* bath ankylosing spondylitis disease activity index, *BASFI* bath ankylosing spondylitis functional index, *EQ*-*5D* EuroQol five-dimensional questionnaire

Table 2 presents the bivariate correlation coefficients between total scores and domains of the EQ-5D-5L and BASDAI/BASFI scores. Generally, there appeared to be ‘high’ (negative) and significant correlation between EQ-5D and BASDAI and BASFI scores (− 0.7897 and − 0.8016 respectively, *p* < 0.0001). The correlation was negative because better scores are indicated by higher EQ-5D and lower BASDAI/BASFI scores. Correlations between individual EQ-5D domains and BASDAI and BASFI were ‘moderate’ to ‘high’ (positive) and significant (range 0.4351 to 0.7924, *p* < 0.0001). The domain correlations were positive because better scores are indicated by lower BASDAI/BASFI scores and lower EQ-5D domain level responses. From a qualitative perspective, the mobility, usual activities, and self-care domains on the EQ-5D-5L would appear to look the most similar/most closely related to the BASFI 10 questions. For the BASDAI 10 questions the pain and discomfort EQ-5D-5L domain look the most similar to each other. Notably, neither of the BASDAI/BASFI instruments include questions related to mental health similar to the anxiety and depression domain of the EQ-5D-5L (the lowest correlation for both BASDAI/BASFI).

**Table 2:** Spearman’s correlation coefficients between EQ-5D-5L (total score and 5 domains), BASDAI, and BASFI scores

	EQ-5D-5L total score	EQ-5D-5L domains				
		Mobility	Self-care	Usual activities	Pain/discomfort	Anxiety/depression
BASDAI score	− 0.7897	0.6577	0.5776	0.7128	0.7924	0.5010
BASFI score	− 0.8016	0.7610	0.7224	0.7628	0.6916	0.4351

Total sample correlations. All correlation coefficients were statistically significant at *p* < 0.0001

*BASDAI* bath ankylosing spondylitis disease activity index, *BASFI* bath ankylosing spondylitis functional index, *EQ*-*5D* EuroQol five-dimensional questionnaire

#### Linear model

The best linear model included squared terms as explanatory variables. In terms of model fit (Table 3), the linear model had generally poorer performance, for instance it had higher RMSE and MAE compared to other model specifications, although notably it had the lowest ME and it performed slightly better than the mixture models with two components (see below). The linear model showed characteristic underestimation of the observed mean EQ-5D values at the extremes of the distribution of disease severity in both plots (Fig. 3 for BASDAI/BASFI).

**Table 3:** Model performance for the response mapping and mixture models (*n* = 5122)

Model type (regression specification)	Explanatory variables	Mean ± SD	Log likelihood	AIC	BIC	ME	MAE	RMSE	ME rank	MAE rank	RMSE rank	Overall rank
Linear OLS	+ squared terms	0.69298 ± 0.21674	2915.3	− 5816.6	− 5770.81	0.00000	0.10065	0.13695	1	12	13	26
Response mappings												
OPROBIT	Main effects	0.69301 ± 0.21294	− 22343.49	44756.98	44985.92	0.00004	0.09893	0.13637	3	8	8	19
OPROBIT	+ squared terms	0.69328 ± 0.21476	− 22,264.05	44628.09	44955.15	0.00001	0.09811	0.13507	2	7	6	15
GOPROBIT	Main effects	0.69250 ± 0.21613	− 22201.27	44562.54	45085.85	− 0.00047	0.09809	0.13539	5	6	7	18
GOPROBIT	+ squared terms	Many negative probabilities were predicted and so the model was considered unreliable and not considered further										
Mixture models												
ALDVMM-2 gap	Main effects	0.68476 ± 0.20025	2177.36	− 4338.72	4286.39	− 0.00821	0.10764	0.14575	14	14	14	42
ALDVMM-3 gap	Main effects	0.69357 ± 0.21367	3660.96	− 7289.96	− 7185.26	0.00060	0.09908	0.13672	8	11	11	30
ALDVMM-4 gap	Main effects	Model convergence not achieved										
ALDVMM-2 gap	+ squared terms	0.68860 ± 0.21287	2513.87	− 5005.74	− 4933.79	− 0.00438	0.10113	0.13688	13	13	12	38
ALDVMM-3 gap	+ squared terms (age, basfi)	0.69372 ± 0.21692	3731.80	− 7423.60	− 7292.77	0.00075	0.09659	0.13362	11	5	5	21
ALDVMM-4 gap	+ squared terms	Model convergence not achieved from adding squared terms										
ALDVMM-2 no gap	Main effects	0.69367 ± 0.21385	3458.52	− 6891.03	− 6806.00	0.00069	0.09900	0.13657	9	9	9	27
ALDVMM-3 no gap	Main effects	0.69367 ± 0.21385	3458.52	− 6885.03	− 6780.37	0.00069	0.09900	0.13657	9	9	9	27
ALDVMM-4 no gap	Main effects	0.69332 ± 0.21820	3601.43	− 7154.86	− 6997.87	0.00035	0.09596	0.13340	4	2	4	10
ALDVMM-2 no gap	+ squared terms	0.69377 ± 0.21672	3563.23	− 7088.43	− 6964.15	0.00080	0.09612	0.13339	12	4	3	19
ALDVMM-3 no gap	+ squared terms	0.69346 ± 0.21646	3600.27	− 7156.53	− 7012.63	0.00049	0.09608	0.13337	6	3	2	11
ALDVMM-4 no gap	+ squared terms	0.69355 ± 0.21624	3640.40	− 7214.80	− 6998.94	0.00057	0.09591	0.13319	7	1	1	9
Observed		0.69298 ± 0.25639										

Note that AIC and BIC are not comparable between response and ALDVMMs. The assessment of the performance of the mapping models based on their ME, MAE, RMSE were ranked with numbers ‘1’indicting the closest fit to observed data. Note. All models with a ‘gap’ (2–4 components) include a truncation at the best possible health state other than full health (i.e., at EQ-5D -5L value = 0.95; health states 12111 or 11211). A one component model with a gap still reflects the gap found in EQ-5D. All models with ‘no gap’ estimate models without a gap, that is, a mixture of Tobit models [47].

*BASDAI* bath ankylosing spondylitis disease activity index, *BASFI* bath ankylosing spondylitis functional index, *ME* mean error, *MAE* mean absolute error, *RMSE* mean absolute error, *AIC* Akaike information criterion, *BIC* Bayesian information criterion

#### Response models

**Fig. 2:**
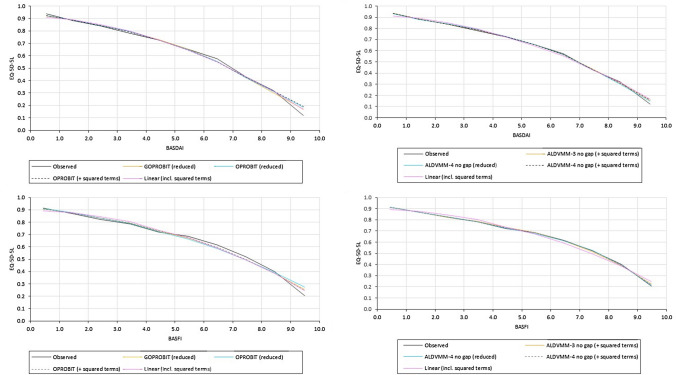
Mean EQ-5D-5L values vs BASDAI score and BASFI score for observed versus predicted data. BASDAI, Bath Ankylosing Spondylitis Disease Activity Index; BASFI, Bath Ankylosing Spondylitis Functional Index; EQ-5D-5L, EuroQol five-dimensional questionnaire. The BADAI and BASFI plots are constructed based on 10 classes/groups (total *n* = 5122). For the BASDAI plot the number of observations contained in each group from the lowest to highest BASDI score are: 410, 696, 752, 577, 599, 584, 602, 470, 275, and 157. For the BASFI plot the number of observations contained in each group from the lowest to highest BASFI score are: 659, 763, 599, 511, 453, 478, 439, 422, 421, and 377

We estimated two response mappings to the EQ-5D-5L, using ordered probit (OPROBIT) and generalised ordered probit (GOPROBIT) models. Table 3 presents the model fit statistics. Overall, the ME and RMSE are smaller in the OPROBIT model (including the explanatory variables BASDAI, BASFI, and age plus squared terms) compared to the GOPROBIT model (reduced). It is notable that the GOPROBIT model (including squared terms) generated a large number of negative predictive probabilities for some domain responses, and although the total sum of probabilities across the 5- response levels equalled 1.0, this model specification was considered unreliable and so was not considered further. On the other hand the GOPROBIT model (reduced) has a lower MAE and AIC (but a higher BIC). Figure 2 shows mean predicted versus mean observed values for response mappings using the overall BASDAI/BASFI scores. There is not much to choose between the different response mapping model types/specifications. All of the response mappings appear to produce slightly higher values at higher BASDAI/BASFI levels when patients are in poorer/worse health. On the other hand, all response mappings, to some degree tend to slightly underestimate values at lower BASDAI/BASFI levels as seen by the BASDAI plot. This is also apparent for mid- to mid-upper range BASDA/BASFI levels, as seen by the BASFI plot for scores in the range 5 to 8.

#### Adjusted limited dependent variable models

We attempted to estimate ALDVMMs with between two and four components and using BASDAI/BASFI overall scores both within the components and as determinants of component membership. We also explored model specifications modelling the gap/truncation point between full health and the next feasible value (i.e., 0.950) included/excluded. Table 3 presents comparisons of the summary measures of the fit statistics. The model with three components, with the gap between full health and the next feasible value included along with squared terms performed the best when considering AIC and BIC statistics. However, model specifications with three and four components that did not include this gap have lower error measures (in terms of ME, MAE and RMSE) than three component reduced models including this gap. The three and four components ALDVMMs including squared terms (followed by the four components reduced model) with no truncation point also outperforms the best performing response mapping models (OPROBIT including squared terms) with better rankings on MAE, RMSE criteria. To give a visual sense of model fit for the respective models, plots of the estimated/predicted means of the best performing ALDVMM models compared with the observed mean across the range of BASFI and BASDAI severity are shown in Fig. 2. The four component model including squared terms and no truncation point appears to predict the observed data a little better than the four component ALDVMM (reduced and no gap) and the three component model (including squared terms and no gap) at most of the BASDAI/BASFI scores– though all models tend to overestimate values for individuals scoring extremely poor values on the BASDAI/BASFI instrument. All model specifications that formally included the gap between full health and the next possible health state value performed somewhat less well compared to those that did not.

**Fig. 3:**
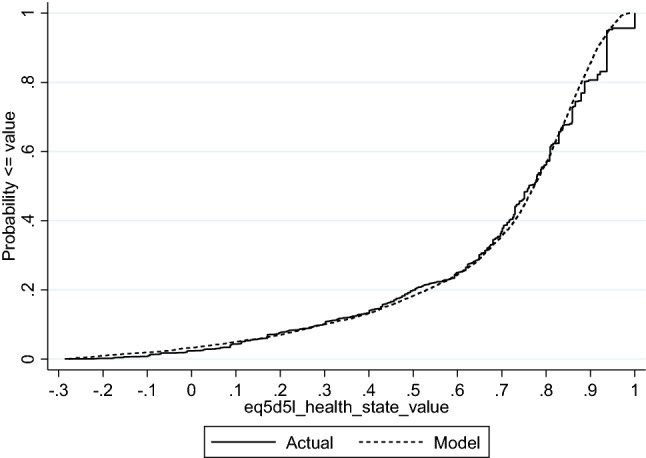
Cumulative distribution function of observed and simulated EQ-5D-5L index of the preferred 4 component mixture model (+ squared terms, no gap)

Figure 3 displays the CDF for the best performing mixture model (and the preferred model overall), the ALDVMM-4 (including squared terms) model without a gap.

Overall, while the simulated data closely aligned to the original data that was distributed over most of the EQ-5D range, the simulated data that were equal to 1 were however unrepresented. This had the impact of a slightly lower estimated mean EQ-5D 0.686 compared to the observed mean of 0.693. The variance was mostly preserved however and was 0.068 in the simulated data versus 0.066 in the original data. This use of simulated data compared with observed data shows that the preferred mapping model can appropriately reflect uncertainty in the majority of utility responses well, however the uncertainty in the upper end of the EQ-5D (responses at full health) is estimated less accurately. Therefore whilst the use of the best ALDVMM mapping models can populate health states for a decision-analytic model with a good degree of accuracy, the algorithm might risk somewhat undervaluing/underestimating EQ-5D values at full health (1.0). This finding is different from previous studies and might in part be due to the smaller number of values equal to 1 found in the observed data. For example, the proportion of observations at full health has been reported in the range 14% to 30% [e.g., 25, 27, 30, 48].

The model fit statistics in Table 3 also suggest that the﻿ ALDVMMS (with either three or four components including squared terms, or four-component reduced with no gap) outperform the best performing response mappings using GOPROBIT (reduced) model or OPROBIT (including squared terms) (e.g., lowest RMSE).

The estimated parameters for the best ALDVMM models and response mappings are provided in the Supplementary Materials (Tables 1 and 2). The preferred ALDVMM and best response mapping algorithms estimated in this study can be implemented via Excel and these are provided as a spreadsheet tool in the Supplementary Materials Spreadsheet File 1.

## Discussion

There are few published studies in axSpA that provide direct estimates of HRQoL from generic preference-based instruments such as the EQ-5D. Such estimates are critical to provide accurate information relating to the cost-effectiveness of new medicines or other interventions. An alternative solution that is extensively employed is the use of mapping from one measure, such as the BASDAI and BASFI, to a utility-based measure such as the EQ-5D.

The current study provides an easy to apply mapping algorithm for the best performing mixture model (four components and squared terms) which had acceptable goodness-of-fit (AIC/BIC) and precision (MAE, RMSE), allowing its use to predict utilities of the more recently developed EQ-5D-5L from the specific BASDAI/BASFI instruments in patients with AS. The MAE (0.09591), RMSE (0.13319) indicated model ALDVMM-4 (with squared terms and no gap) to provide the best fit of the data, the AIC was however higher than both ALDVMM-3 models that did not formally include this gap (Table 3: − 7215 vs − 7423 and − 7290, respectively). In all models the predicted errors were not uniform across the range of the EQ-5D-5L. Larger errors were generally most apparent for utilities reflecting the poorest health states (Fig. 2 plot of predicted versus observed values).

With the increasing evidence that mapping methods that are developed from linear regression models exhibit inferior statistical properties than those developed from alternative methods such as mixture models [25–29, 33], our study adds to the existing evidence base by comparing the statistical performance of response mapping models with ALDVMM mixture models. Using new primary data from a large longitudinal registry study of axSpA patients in the UK [39], we show that the relationship between EQ-5D-5L and BASDAI/BASFI responses can be effectively estimated using ALDVMM mixture models and response mapping models. Based on statistical fit criteria (Table 3), the response mapping models broadly offered comparable predictive performance to the mixture models, providing a reasonable fit relative to the observed EQ-5D-5L data.

Specifically, however, for the reasons outlined above, we suggest the four-component ALDVMM mixture model (including squared terms) is the preferred model with best overall fit to the EQ-5D-5L data (vs either the three-component with squared terms or four-component reduced models). Other mapping studies have been conducted using BASDAI/BASFI outcomes, however, these focused only on the estimation of EQ-5D-3L from BASDAI/BASFI scores [21, 27, 35] and only one investigated the performance of models other than linear regression using mixture models and response mappings [27].

There are some potential limitations to our study that should be considered. First, it would be preferable to assess the generalisability of the algorithms in another independent dataset [46], but such external data were not available when conducting this study. Further validation of our study results on external data sets could be conducted when such data sets become available.

Second, the dataset consists of observations from the entire UK, but tariffs from England are used [42] for the EQ-5D-5L. Research is ongoing to produce a UK wide value set for the EQ-5D-5L and will address the question of whether preferences of the UK population (i.e., including Scotland, Wales, and Northern Ireland) are consistent with values from England. Moreover, research is ongoing to create a robust EQ-5D-5L value set for use in the UK that NICE will recommend for future use in HTAs. It is therefore important to recognise that future EQ-5D-5L values may be subject to change, and that further research may be required once these values become available [49]. We note that mixture modelling between BASDAI/BASFI and EQ-5D-5L using the current valuation set for England offers a good model fit, but changes to valuation would change the parameters of this mapping model. Therefore, we also draw attention to the potential further usefulness of the response mapping algorithms, which are not dependent on any one country-specific value set.

Third, although the data used in this study span the full range of the BASDAI/BASFI and (majority) of the EQ-5D-5L scores, there are fewer observations at the higher end of the BASDAI/BASFI and EQ-5D-5L as well as the lower end of both instruments, although this is a limitation common to many mapping studies [27]. Fourth, although the sample size used to develop the mapping algorithms was relatively large (*n* = 5122), it may have affected the response mappings more as they require observations at all five levels of the EQ-5D domains. However, only a very small number of patients were choosing the ‘extreme problems’ level, which could potentially bias the estimation of the parameters and further limit the (response) model performance at these data points.

Weighed against this, in cohort decision models with health states not located at the extreme of poor health, bias from mapping would constitute only a negligible effect on estimated cost-effectiveness [44]. In individual patient-level simulations, it is recommended that uncertainty in the estimated mapping coefficients and their correlations is incorporated through probabilistic sensitivity analysis using the variance–covariance matrices and that individual-level variability is considered using the distributions of error terms – both provided in the excel tool. (and Supplementary Table 3).

Our study has several strengths. First, in addition to the more widely used direct mapping, this study has also conducted indirect response mapping to predict responses to each of the EQ-5D dimensions. A key advantage of this approach is that it allows different EQ-5D value sets to be applied, thus the reported mapping functions can be more widely applied by users from other countries. However, it should be noted that the generalisability of the indirect response mapping functions depends on whether axSpA patients from other countries will have a similar response pattern to patients in the UK. Further external validation is warranted. Second, the data are derived from a non-trial “real-world” sample that is likely to be nationally representative for most patients with axSpA in the UK. Furthermore, the sample size is also bigger than any previous mapping study in this population (and included participants from three nations in the UK-England, Wales, and Scotland instead of only one UK nation –Wales) [27]. Thus, the mapping algorithms developed in this study are likely to be generalisable to other axSpA patients.

The relationship between BASDAI/BASFI and EQ-5D-3L for use in axSpA has been considered in only a limited number of previous studies, most notably by Wailoo et al. 2015 [27], who found that response mapping and mixture models outperformed linear regression. However, to our knowledge no previous mapping study has researched the relationship between BASDAI/BASFI and EQ-5D-5L utilities. Our study is therefore the first that attempts to provide estimates for the calculation of the EQ-5D-5L as a function of BASDAI/BASFI in patients with axSpA when analysts have access only to BASDAI/BASFI scores of axSpA patients.

## Conclusions

To our knowledge, this is the first study to develop mapping algorithms from the widely used BASDAI/BASFI measures to EQ-5D-5L utility values in patients with axSpA. The results showed that mixture models, and to a slightly lesser degree, response mapping provided reliable algorithms for predicting EQ-5D-5L utilities from BASDAI/BASFI scores. These algorithms can be used in applied cost-effectiveness analysis in axSpA where EQ-5D-5L is the target outcome of interest.

Further research using mixture models and different datasets, for example using data from national axSpA registries that include the EQ-5D could help to further develop and validate these mapping algorithms in patients with axSpA (by further pooling other larger datasets for instance). Other techniques in the mapping literature such as beta-binomial regression models might also be explored in future research. In addition, the indirect mapping instrument functions reported in this study for the EQ-5D-5L will further facilitate the calculation of EQ-5D-5L utility scores using other country-specific value sets. A user-friendly freely accessible Excel tool has been provided to assist analysis with the implementation of the best performing ALDVMM mapping algorithms and best performing response mapping model (available in the Appendix, Supplemental Material). Although it remains preferable to have health utilities data derived directly from administering the EQ-5D-5L, the mapping algorithms developed in this study can be used to inform the generation of reliable health utility estimates (from BASDAI/BASFI scores) in cost-effectiveness analyses of interventions for axSpA (e.g., clinical studies or to populate model-based economic evaluations), when only responses from the disease specific BASDAI/BASFI instruments have been collected.

## Supplementary Information

Below is the link to the electronic supplementary material.

Supplementary file1 (DOCX 1029 KB)

Supplementary file2 (XLSX 262 KB)

## Declarations

### Precis

Mixture models and response mapping can be used to crosswalk between BASDAI/BASFI and EQ-5D-5L to provide inputs for applied cost-effectiveness studies evaluating treatments in AxSpA.
