# Supplementary material for: Generating EQ-5D-5L health utility scores from BASDAI and BASFI: a mapping study in patients with axial spondyloarthritis using longitudinal UK registry data
Source: Eur J Health Econ. 2022 Feb 3;23(8):1357–69. doi: 10.1007/s10198-022-01429-x (PMC9550731; doi:10.1007/s10198-022-01429-x)
Supplement: Supplementary file 1 — Supplementary file1 (DOCX 1029 KB) [file 10198_2022_1429_MOESM1_ESM.docx]

**SUPPLEMENTARY MATERIAL**

**Supplementary Table 1: Mixture models estimates**

|  | **ALDVMM-4***  **(including squared terms with no truncation point formally modelled)** | | | **ALDVMM-4***  **(reduced explanatory terms with no truncation point formally modelled)** | | |
| --- | --- | --- | --- | --- | --- | --- |
|  | **Parameter** | **Robust Std. Err.** | **P-value** | **Parameter** | **Robust Std. Err.** | **P-value** |
| **Component 1** |  |  |  |  |  |  |
| BASDAI | -.0327073 | .0029962 | 0.000 | -.0238472 | .0010309 | 0.000 |
| BASFI | -,0228277 | .0023068 | 0.000 | -.0175899 | .0009442 | 0.000 |
| Age | .0006631 | .0006095 | 0.277 | .0006469 | .0001055 | 0.000 |
| BASDAI^2^ | .001997 | .0003772 | 0.000 |  |  |  |
| BASFI^2^ | -.000105 | .0002712 | 0.699 |  |  |  |
| Age^2^ | -.7.37e-07 | 5.82e-06 | 0.899 |  |  |  |
| Constant | .9401599 | .0161423 | 0.000 | .9192235 | .0055518 | 0.000 |
| **Component 2** |  |  |  |  |  |  |
| BASDAI | 0 | (omitted) |  | 0 | (omitted) |  |
| BASFI | 0 | “ |  | 0 | “ |  |
| Age | 0 | “ |  | 0 | “ |  |
| BASDAI^2^ | 0 | “ |  |  |  |  |
| BASFI^2^ | 0 | “ |  |  |  |  |
| Age^2^ | 0 | “ |  |  |  |  |
| Constant | 2.881103 | . | . | 2.81103 | . | . |
| **Component 3** |  |  |  |  |  |  |
| BASDAI | -.0407092 | .0242324 | 0.093 | -.0524457 | .0223972 | 0.019 |
| BASFI | .1283773 | .0273751 | 0.000 | -.0398263 | .0167706 | 0.018 |
| Age | .0103805 | .0029057 | 0.000 | .0021316 | .0014404 | 0.139 |
| BASDAI^2^ | -.0002512 | .0017763 | 0.888 |  |  |  |
| BASFI^2^ | -.0145201 | .0018818 | 0.000 |  |  |  |
| Age^2^ | -.0000662 | .000028 | 0.018 |  |  |  |
| Constant | .2068678 | .1279977 | 0.106 | .8304059 | .3051768 | 0.007 |
| **Component 4** |  |  |  |  |  |  |
| BASDAI | -.0210227 | .0163791 | 0.199 | -.0163391 | .0025628 | 0.000 |
| BASFI | -.0431581 | .0128183 | 0.001 | -.0090952 | .0026372 | 0.001 |
| Age | .0041423 | .0018177 | 0.023 | .0005458 | .0004093 | 0.182 |
| BASDAI^2^ | .0012726 | .0013226 | 0.336 |  |  |  |
| BASFI^2^ | .0023787 | .0014372 | 0.098 |  |  |  |
| Age^2^ | .6408449 | .0000209 | 0.009 |  |  |  |
| Constant | .6408449 | .0659918 | 0.000 | .6046162 | .0272104 | 0.000 |
| **Probability Component 1** |  |  |  |  |  |  |
| BASDAI | -.71257 | .0843827 | 0.000 | -.1398833 | .1260893 | 0.267 |
| BASFI | -.0426675 | .0640524 | 0.505 | -.5967924 | .1065161 | 0.000 |
| Constant | 6.741222 | .4302469 | 0.000 | 6.267529 | .4497584 | 0.000 |
| **Probability Component 2** |  |  |  |  |  |  |
| BASDAI | -6.654394 | 1.037884 | 0.029 | -.6314622 | .2423698 | 0.009 |
| BASFI | -.5231506 | .2394822 | 0.029 | -6.089481 | .9156079 | 0.000 |
| Constant | 8.308282 | .594894 | 0.000 | 7.91423 | .5788729 | 0.000 |
| **Probability Component 3** |  |  |  |  |  |  |
| BASDAI | -.1686247 | .1442858 | 0.140 | .4303207 | .1154429 | 0.000 |
| BASFI | .4805228 | .0805826 | 0.000 | -.022555 | .280567 | 0.936 |
| Constant | -.1699447 | .8765687 | .0846 | -1.928903 | 3.754105 | 0.607 |
| /lns_1 | -2.62994 | .0168702 | 0.000 | -2.594364 | .0289247 | 0.000. |
| /lns_2 | -49.64091 | . | . | -49.64091 | . | . |
| /lns_3 | -1.702265 | .0220146 | 0.000 | -1.818447 | .2697724 | 0.000 |
| /lns 4 | -3.43162 | .1921745 | 0.000 | -3.23301 | .2813787 | 0.000 |
| **Component variance** |  |  |  |  |  |  |
| sigma1 | .0720828 | .0012161 |  | .0746934 | .0021605 |  |
| sigma2 | 2.76e-22 | . |  | 2.76e-22 | . |  |
| sigma3 | .1822702 | .0040126 |  | .1622776 | .043778 |  |
| sigma 4 | .0323345 | .0062139 |  | .0394386 | .0110972 |  |

*Model estimates using the aldvmm ‘inim(cons)’ option to estimate a constant only model first and use the estimated parameters in the full model specification. [49]

**Supplementary Table 2: Response mapping models estimates**

**Ordered probit (OPROBIT) including squared terms**

|  | **Mobility** | | | **Self-care** | | | **Usual Activities** | | | **Pain and Discomfort** | | | **Anxiety and Depression** | | |
| --- | --- | --- | --- | --- | --- | --- | --- | --- | --- | --- | --- | --- | --- | --- | --- |
|  | Parameter | Robust Std Err. | P-value | Parameter | Robust Std Err. | P-value | Parameter | Robust Std Err. | P-value | Parameter | Robust Std Err. | P-value | Parameter | Robust Std Err. | P-value |
| BASDAI | 0.2356848 | 0.0342565 | 0.000 | 0.0398732 | 0.041939 | 0.342 | 0.3090673 | 0.3020104 | 0.000 | 0.5892901 | 0.03691201 | 0.2306551.000 | 0.2306551 | 0.0326211 | 0.000 |
| BASFI | 0.2078193 | 0.0290754 | 0.000 | 0.2009675 | 0.0363538 | 0.000 | 0.2500807 | 0.0279687 | 0.000 | 0.082494 | 0.288276 | 0.000 | 0.0300857 | 0.0277884 | 0.279 |
| Age | -0.0313015 | 0.0073047 | 0.000 | 0.0002391100.0.0002391 | 0.0086212 | 0.978 | -0.0100238 | 0.0075768 | 0.186 | -0.0073147 | 0.0076612 | 0.340 | -0.0068887 | 0.000748903 | 0.358 |
| BASDAI^2^ | -0.0110979 | 0.0033534 | 0.001 | 0.0007134 | 0.0038824 | 0.854 | -0.0098646 | 0.0031652 | 0.002 | -0.0117933 | 0.00348405 | 0.001 | -0.0050989 | 0.0033352 | 0.126 |
| BASFI^2^ | 0.0167081 | 0.0028471 | 0.000 | 0.024997 | 0.0033212 | 0.000 | 0.0086977 | 0.0027346 | 0.001 | 0.0072249 | 0.026966 | 0.007 | 0.0071404 | 0.0026558 | 0.007 |
| Age^2^ | 0.0003165 | 0.000071 | 0.000 | -0.000071 | 0.0000851 | 0.404 | 0.000043 | 0.0000741 | 0.562 | 0.00000727 | 0.000074000003 | 0.922 | -0.0000766 | 0.0000732 | 0.295 |
| /cut1 | 0.7686018 | 0.1911341 |  | 1.914565 | 0.2249096 |  | 1.104516 | 0.1953674 |  | -0.2354611 | 0.1958659 |  | 0.4514973 | 0.1929625 |  |
| /cut2 | 2.068747 | 0.01928935 |  | 3.154426 | 0.2274852 |  | 2.712919 | 0.1996385 |  | 2.224749 | 0.2020920007 |  | 1.489194 | 0.1936575 |  |
| /cut3 | 3.572934 | 0.1954715 |  | 4.669232 | 0.2342275 |  | 4.203888 | 0.203409 |  | 4.11397 | 0.2076707 |  | 2.579714 | 0.1961589 |  |
| /cut4 | 5.591492 | 0.2223592 |  | 6.014359 | 0.263289 |  | 5.593201 | 0.2122167 |  | 5.705866 | 0.2111677 |  | 3.351324 | 0.200062 |  |

**Generalised ordered probit (GOPROBIT) reduced**

|  | **Mobility** | | | **Self-care** | | | **Usual Activities** | | | **Pain and Discomfort** | | | **Anxiety and Depression** | | |
| --- | --- | --- | --- | --- | --- | --- | --- | --- | --- | --- | --- | --- | --- | --- | --- |
|  | Parameter | Robust Std Err. | P-value | Parameter | Robust Std Err. | P-value | Parameter | Robust Std Err. | P-value | Parameter | Robust Std Err. | P-value | Parameter | Robust Std Err. | P-value |
| **Level 1** |  |  |  |  |  |  |  |  |  |  |  |  |  |  |  |
| BASDAI | .132063 | .0146053 | 0.000 | .0117127 | .0151958 | 0.441 | .2133212 | .0157621 | 0.000 | .4997034 | .0529381 | 0.000 | .1723556 | .0124649 | 0.000 |
| BASFI | .3491795 | .0140185 | 0.000 | .4489578 | .0149314 | 0.000 | .3276745 | .0159059 | 0.000 | .0806331 | .0281202 | 0.004 | .0770495 | .0106697 | 0.000 |
| Age | -.004729 | .001673 | 0.005 | -.0086095 | .001689 | 0.000 | -.0044658 | .0017431 | 0.010 | .0025636 | .0025636 | 0.713 | -.0124426 | .0014187 | 0.000 |
| Constant | -1.270587 | .094382 | 0.000 | -2.034503 | .0992633 | 0.000 | -1.215326 | .0977251 | 0.000 | .1486404 | .1486404 | 0.932 | -.2874909 | .0791269 | 0.000 |
| **Level 2** |  |  |  |  |  |  |  |  |  |  |  |  |  |  |  |
| BASDAI | .1387232 | .0152547 | 0.000 | .0951127 | .0184297 | 0.000 | .2185695 | .0151232 | 0.000 | .0172332 | .0172332 | 0.000 | .1969279 | .0142757 | 0.000 |
| BASFI | .3737288 | .0139557 | 0.000 | .5060696 | .0220772 | 0.000 | .3406695 | .0136001 | 0.000 | .012865 | .012865 | 0.000 | .1262782 | .0121494 | 0.000 |
| Age | .0044223 | .0017655 | 0.012 | -.004303 | .0020496 | 0.036 | -.0069847 | .0017649 | 0.000 | .0018002 | .0018002 | 0.000 | -.0159007 | .001569 | 0.000 |
| Constant | -3.173077 | .1187887 | 0.000 | -4.309735 | .1828083 | 0.000 | -2.77384 | .116053 | 0.000 | .1045039 | .1045039 | 0.000 | -1.506745 | .0970548 | 0.000 |
| **Level 3** |  |  |  |  |  |  |  |  |  |  |  |  |  |  |  |
| BASDAI | .1317997 | .0217137 | 0.000 | .0666559 | .0280131.0 | 0.017 | .2228524 | .021847 | 0.000 | .4529724 | .0213606 | 0.000 | .1941243 | .0223311 | 0.000 |
| BASFI | .4195153 | .0293082 | 0.000 | .6229501 | .0781359 | 0.000 | .3677816 | .026572 | 0.000 | .1949853 | .0157325 | 0.000 | .1262782 | .019502 | 0.000 |
| Age | .0067141 | .0024193 | 0.006 | -.0093523 | .0035662 | 0.009 | -.0065383 | .002409 | 0.007 | -.0125915 | .0020994 | 0.000 | -.0159007 | .0025106 | 0.000 |
| Constant | -5.068094 | .249562 | 0.000 | -6.308641 | .692226 | 0.000 | -4.509014 | .2106818 | 0.000 | -3.853497 | .1569114 | 0.000 | -1.506745 | .1565251 | 0.000 |
| **Level 4** |  |  |  |  |  |  |  |  |  |  |  |  |  |  |  |
| BASDAI | -.0260639 | .0500149 | 0.602 | .1325467 | .086526 | 0.126 | .1324254 | .0311799 | 0.000 | .4547525 | .0392521 | 0.000 | .1462475 | .0430541 | 0.001 |
| BASFI | .3436528 | .0804092 | 0.000 | .3031935 | .1577398 | 0.055 | .2364223 | .0327813 | 0.000 | .1613224 | .0368412 | 0.000 | .1363539 | .0322229 | 0.000 |
| Age | .0050849 | .0066957 | 0.448 | .0000383 | .0083232 | 0.996 | -.0021388 | .0035334 | 0.545 | -.088736 | .0033391 | 0.008 | -.0182429 | .0036789 | 0.000 |
| Constant | -5.165029 | .7881308 | 0.000 | -5.805045 | .8181761 | 0.000 | -4.386395 | .3315397 | 0.000 | -5.429889 | .3400706 | 0.000 | -3.011084 | .2958606 | 0.000 |

**Supplementary Table 3:** Covariance matrix for Mixture models and Response mapping models

1. ALDVMM-4 (including squared terms with no truncation point formally modelled)

2. ALDVMM-4 (reduced explanatory terms with no truncation point formally modelled)

3. ALDVMM-3 (including squared terms with truncation point formally modelled)

4. Response mapping (OPROBIT including squared terms)

**1. ALDVMM-4 (including squared terms with no truncation point formally modelled)**

**2. ALDVMM-4 (reduced explanatory terms with no truncation point formally modelled)**

**3. ALDVMM-3 (including squared terms with truncation point formally modelled)**

**4. Response mappings**

**(OPROBIT including squared terms)**

Mobility

Self-care

Usual activities

Pain/Discomfort

Anxiety/Depression

**GOPROBIT (main effects only)**

Mobility

Self-care

Usual activities

Pain/Discomfort

Anxiety/Depression
